# LiVE-STROKE: live video from bystander to the medical dispatcher in potential stroke cases

**DOI:** 10.1186/s13049-026-01559-8

**Published:** 2026-02-03

**Authors:** E. Rischel, H. K. Iversen, H. Christensen, J. Wenstrup, H. C. Christensen, G. Linderoth

**Affiliations:** 1 Copenhagen Emergency Medical Services, Telegrafvej 5, opg. 2, 3rd Floor, Ballerup, Denmark; 2 Department of Neurology, Rigshospitalet, University Hospital of Copenhagen, Copenhagen, Denmark; 3 Department of Neurology, Bispebjerg Hospital, University Hospital of Copenhagen, Copenhagen, Denmark; 4 Prehospital Centre, Region Zealand, Næstved, Denmark; 5 Department of Brain and Spinal Cord Injuries, Rigshospitalet Glostrup, University Hospital of Copenhagen, Copenhagen, Denmark; 6 Department of Anaesthesia and Intensive Care, Bispebjerg Hospital, University Hospital of Copenhagen, Copenhagen, Denmark

**Keywords:** Stroke, Medical dispatcher, Live video, Emergency medical services, Neurological exam

## Abstract

**Background:**

Identification of stroke during an emergency call remains challenging. We aimed to explore performed stroke examination with prehospital live video transmission during an emergency call from a bystander’s smartphone to the medical dispatcher.

**Methods:**

Since 2019, the Emergency Medical Services (EMS) in Copenhagen, consisting of the Emergency Medical Coordination Centre (EMCC) and the Out-of-Hours Health Service (OOHS), have enabled medical dispatchers to add live video to emergency calls. Dispatchers are registered nurses or paramedics. Live video is initiated via text message link sent to the caller’s smartphone. We included all cases where the dispatcher added live video to the emergency call and stroke was suspected or later identified according to the Danish Stroke Registry. The video-recorded on-site stroke examinations were retrospectively analyzed. No specific guidance for video-based stroke examination was in place.

**Results:**

A successful live video transmission was achieved in 550 potential stroke cases, where 52% were correctly examined for facial paresis and 16% for both facial and extremities paresis. In six patients, hemiparesis was observed without an examination. The group with ‘considered stroke’ had the highest amount of examined cases. Dispatchers suspected stroke in 450 cases, and stroke was confirmed in 16% (*n* = 73). Stroke was diagnosed in 126 cases and was not suspected in 46% of these cases (missed stroke *n* = 63, TIA *n* = 10). Patients with missed stroke had significantly less accurate examinations and more often hemorrhagic stroke, compared to patients with confirmed stroke.

**Conclusion:**

Video is not routinely used for stroke assessment during emergency calls, and only a small number of cases are sufficiently examined for stroke with video. Implementation of a structured protocol for live video guided stroke examinations during emergency calls could be beneficial but best practice is still unknown.

## Introduction

Stroke is the fourth leading cause of death and the leading cause of disability in Denmark [1], and the cause of significant socio-economic costs [2, 3]. Furthermore stroke is the third leading cause of disability-adjusted life years, a measure for loss of life years due to early death or years living with disability [4]. Stroke is either ischemic (85%) or haemorrhagic (15%). In ischemic stroke the reperfusion treatments are effective but highly time-dependent [5–8] and only a small proportion of patients (below 25%) receive reperfusion treatment [9].

EMS Copenhagen in Denmark is organized as an integrated service with 2 individual call lines. The “1813—Medical Helpline” is an out-of-hours service (OOHS) for nonacute cases during hours where general practitioners are inaccessible, and the “1–1–2 – Emergency Helpline,” (EMCC) for acute emergencies, where all dispatchers are trained nurses or paramedics [10] with additional 6 weeks of training in handling emergency calls [11–13]. At Copenhagen EMS, medical dispatchers recognized stroke with a sensitivity of 64% (Emergency Medical Call Centre, EMCC) and 25% (Out-of-Hours Service, OOHS), and a specificity of 96% (EMCC) and > 99% (OOHS) [11]. The medical dispatchers use Danish Index as a decision-support tool, which includes criteria with corresponding response times based on severity (Danish Index stroke criterion: see Appendix 2) as well as suggested questions addressing motor power in the arms and legs, neglect, and aphasia [14]. However, a standardised tool for stroke evaluation at Copenhagen EMS has not been implemented [12], and since 2019, the opportunity of adding live video to emergency calls has been available. Studies on live video as a diagnostic aid for the medical dispatcher are few and pertain to emergency calls about cardiac arrest. These studies indicate that addition of live video can increase precision in both dispatch decisions [13], prioritisation and resource allocation [15], and bystanders have indicated strong support for video use in emergency calls [16, 17]. Live video as a diagnostic aid for medical dispatchers regarding stroke has, to our knowledge, not been investigated.

We aimed to explore the use of live video to support on-scene neurological assessment conducted by medical dispatchers as part of routine handling of emergency calls. Our analysis included calls in which stroke was initially considered or suspected as well as those in which stroke was not recognised during dispatch but was later diagnosed in hospital, to compare differences in stroke assessment using live video.

## Methods

### Setting

This is a retrospective observational study. The study was conducted at EMS Copenhagen, which covers 2333km^2^ and a population of 1.8 million [18]. In accordance with current Danish neurovascular treatment guidelines, patients with suspected stroke (Danish Index stroke criterion: see Appendix 2) should be triaged as “A”, as fast as possible, resulting in an ambulance with light and sirens [11–13]. If a transient ischemic attack (TIA, symptoms of stroke up to 24 h [19]) is suspected (Danish Index TIA criterion: see Appendix 2) a “B” response with arrival within 30 min is sent. On-scene ambulance personnel evaluate the patient for signs of stroke, confer with an on-call thrombolysis neurologist to assess eligibility for emergency neurological examination and imaging at a stroke centre [20, 21].

All registrations during the emergency call, including categories, emergency response, timestamps for ambulance dispatch, on-scene arrival, departure, and arrival to the hospital, are saved within the dispatch system provided by Logis Solutions and linked to the subjects’ unique civil registration number (CPR, assigned to all Danish residents for administrative use in the public sector, including the health sector) [11]. Since June 2019, the medical dispatcher has had the opportunity to add live video to the emergency call. The medical dispatchers were trained in the addition of live video with prompts for guiding the caller. The medical dispatcher sends a text message to the caller with a smartphone link for access to video transmission. The caller must accept transmission of the video, activate the link, and inform people present. After confirmation, the smartphone begins transmission of a secure video, livestreaming from the scene to the medical dispatcher.

### Data collection

The data were retrieved from two registry cohorts: The dispatch-systems at EMS Copenhagen, and the Danish Stroke Registry (DSR), a nationwide mandatory registry, containing data on patients with stroke admitted to Danish hospitals since 2003 [19]. All strokes diagnosed at a Danish hospital with symptom onset within the last week are registered in DSR with type of stroke (intracerebral hemorrhage, infarction, unspecified, subarachnoid hemorrhage or TIA) and symptoms on arrival at the hospital such as consciousness, eye movements, arm and hand movements, leg movement, orientation, speech, facial paresis, and gait (Scandinavian Stroke Scale: SSS score) [22]. The period for the study was June 2019 until and including December 2023.

The inclusion criteria were emergency calls with live video with a potential stroke or TIA criteria at EMCC (see Appendix 2) or registered as a stroke or TIA in DSR. To include emergency calls where a stroke examination had been provided but not initially assigned stroke or TIA criteria by EMCC, we also included calls with OOHS criteria (see Appendix 2) or free-text descriptions indicating possible stroke symptoms, such as paresis, weakness, and paraesthesia, and where the medical dispatcher during the call considered stroke. The patients were classified as suspected stroke if assigned a stroke code (A.26.03–4) or a free-text indicating stroke suspicion. The patients with stroke had to have called EMS within 72 h of symptom onset (DSR), a cut-off chosen to include patients with stroke that contacted during the weekends closing hours for general practitioners. Furthermore, calls occurring within 24 h prior to onset of symptoms in DSR were included.

Exclusion criteria included: patients younger than 18 years of age; diagnoses of subarachnoid haemorrhages diagnosis registered in DSR due to distinctly different clinical presentation; cases with criteria or free-text indications of possible stroke symptoms where stroke was neither discussed during the emergency call nor registered as stroke in the DSR; technical difficulties during the call that rendered video recording unusable; and storage issues resulting in missing audio or video files.

The following data for the video calls were recorded: Contact pathway (EMCC or OOHS); whether the medical dispatcher assigned a stroke criterion; EMS triage levels A-F, referral to a local emergency room or general practitioner, or no further patient assessment; patient age; and patient sex; Stroke identified at the hospital (registration in DSR). If stroke was diagnosed at the hospital stroke type and patients’ symptoms were registered.

### Video analysis

A medical doctor conducted the analyses of the videos. This process was conducted under the supervision of two senior stroke neurologists with expertise in stroke assessment and thrombolysis workflow and a pre-hospital anaesthesiologist. The following analysis parameters were chosen based on basic stroke assessment such as examining side difference and aphasia, and based on potential challenges such as consciousness, language barriers and poor examination conditions.

The analysed parameters in the code directory (Appendix 1) included:

- ◦ *Whether the medical dispatcher considered stroke during the audio call prior to video*, answered ‘yes’ or ‘no’, based on questions asked by the medical dispatcher, assessed to ascertain whether the addition of video influenced eventual stroke criterion assignment.
- ◦ *Speech difficulties,* assessed for their potential effect on the threshold for assigning a stroke dispatch code. Such impairments may increase the risk of miscommunication, potentially resulting in less precise dispatch coding; however, as direct manifestations of stroke, they may also increase the probability of assigning a stroke criterion at EMS.
- ◦ *Quality of facial and extremity examinations,* assessed to quantify the extent of neurological examination and effective use of live video. This was documented by assessing whether side differences were adequately examined, with possible responses categorized as: yes; incomplete examination; patient does not follow orders; spontaneous movement or motor deficit observed on video without formal examination; or no. To be categorized as a correct examination, the answer had to be ‘yes’ and could for facial paresis include smiling or showing of teeth, and for extremities be Barré test or squeezing of hands simultaneously for arm power and gait or elevation against gravity for leg power.
- ◦ *Patient positioning for motor impairment assessment,* assessed to determine the validity of the examination, as inadequate positioning – such as visualizing only one side of the patient – compromises the ability to assess for lateralized motor deficits.
- ◦ *Consciousness,* assessed as a factor contributing to inadequate examinations, given EMS medical professionals’ limited training in examining unconscious patients for stroke.
- ◦ *Language barriers,* assessed for potential bias affecting these patients, and to assess whether addition of live video mitigated this bias.
- ◦ *Examination conditions,* assessed for potential circumstances that could impede the examination, potentially limiting the usefulness of live video in stroke assessment.

The SSS scores from the DSR were a reference standard for findings that medical dispatchers might have observed if a comprehensive neurological assessment had been conducted during the live video call. It is acknowledged, however, that symptoms may have evolved or regressed between the time of the call and the neurological evaluation performed by the stroke neurologist at the hospital. Patients with transient ischemic attack (TIA) were excluded from the missed stroke group, because symptoms could resolve prior to the emergency call.

### Statistics

The analyses were performed using SAS Enterprise Guide version 7.1 statistical software (SAS Institute Inc., Cary, NC, USA), R (version i386 4.1.1), Rstudio statistical software made by Free Software Foundation, Boston Massachusetts, USA and Microsoft Excel.

The ‘COUNT.IF’ function in Excel was employed to generate matrices for the construction of cumulative bar charts, stratified by examination type and examination quality relative to stroke suspicion, as well as to delineate differences between missed and detected stroke participants within the analysed dataset. A chi-square test of independence was conducted to examine the association between examination of missed and confirmed stroke. Furthermore, the sensitivity for stroke detection in emergency calls with added video was calculated and the positive predictive value. Specificity and negative predictive value could not be calculated, since the true negative value is not known – the data set only includes live video cases with a stroke response, cases where stroke was considered and/or cases where stroke was diagnosed.

## Results

**Fig. 1:**
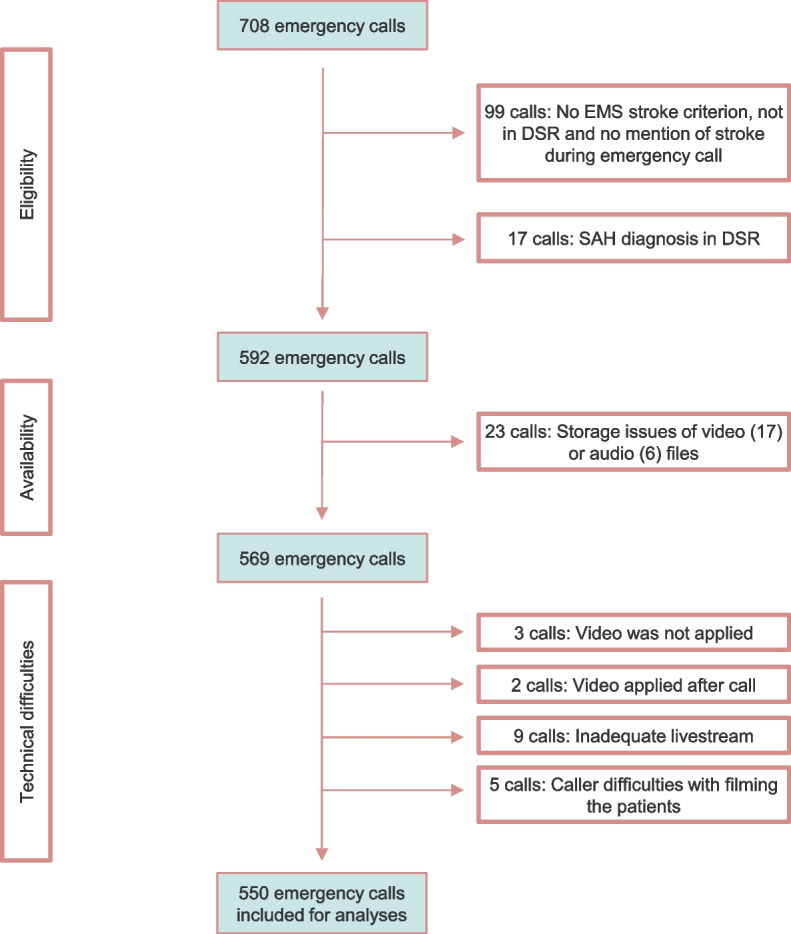
Strobe diagram for exclusion process

A total of 708 emergency calls were initially considered for analysis. Ninety-nine calls were excluded because they were not assigned the stroke criterion from EMS, were not registered in DSR or stroke was not discussed during the call. Seventeen calls were excluded due to SAH diagnosis in DSR. Twenty-three calls (3,2%) were excluded due to storage issues affecting video [17] or audio [6] files. Lastly, 19 calls (2,7%) were excluded due to technical difficulties: 3 where video was not applied; 2 where video was initiated after the call ended; 9 with inadequate video streaming; and 5 with caller difficulties in filming the patient. Consequently, the data set comprised 550 calls (Fig. 1).

**Fig. 2:**
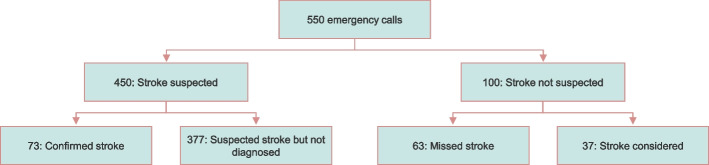
Flow diagram of stroke suspicion and diagnosis for the included emergency calls. *Suspected stroke*: The medical dispatcher activated a stroke response. *Confirmed stroke*: The medical dispatcher suspected stroke and stroke was diagnosed at the hospital. *Stroke not suspected*: The medical dispatcher did not activate a stroke response. *Missed stroke*: The medical dispatcher did not suspect stroke, but stroke was diagnosed at the hospital. *Stroke considered*: The medical professional mentioned stroke or asked about stroke-related symptoms during the call, but a stroke response was not activated

The study population comprised of 550 participants, out of which 136 participants were diagnosed with stroke in hospital (Table 1). Stroke was suspected after the examination in 450 participants and confirmed in 73 participants (16%, confirmed stroke). Stroke was not suspected after the examination in 63 participants but later diagnosed with stroke—missed stroke (TIA *n* = 10). In the 37 remaining participants, stroke was considered during the call before video, but stroke was not suspected after stroke examination, and stroke was not diagnosed subsequently in the hospital (Fig. 2).

**Table 1:** Characteristics of study population

Characteristics	Stroke (*n* = 136)	No stroke (*n* = 414)	Total (*n* = 550)
Call centre			
112 calls	76% (*n* = 103)	44% (*n* = 184)	52% (*n* = 287)
1813 calls	24% (*n* = 33)	56% (*n* = 230)	48% (*n* = 263)
Gender			
Male	53% (*n* = 72)	45% (*n* = 186)	47% (*n* = 258)
Female	47% (*n* = 64)	55% (*n* = 228)	53% (*n* = 292)
Response			
A	71% (*n* = 97)	79% (*n* = 329)	77% (*n* = 426)
B	12% (*n* = 16)	3% (*n* = 11)	5% (*n* = 27)
Other	17% (*n* = 23)	18% (*n* = 74)	18% (*n* = 97)
Age (average 57)			
18–40	9% (*n* = 12)	28% (*n* = 117)	23% (*n* = 129)
41–60	27% (*n* = 37)	32% (*n* = 133)	31% (*n* = 170)
61–80	44% (*n* = 60)	28% (*n* = 114)	32% (*n* = 174)
81 +	20% (*n* = 27)	12% (*n* = 50)	14% (*n* = 77)
Language barrier	10% (*n* = 14)	13% (*n* = 53)	12% (*n* = 67)
Affected state of consciousness	15% (*n* = 21)	7% (*n* = 31)	9% (*n* = 52)

**Fig. 3:**
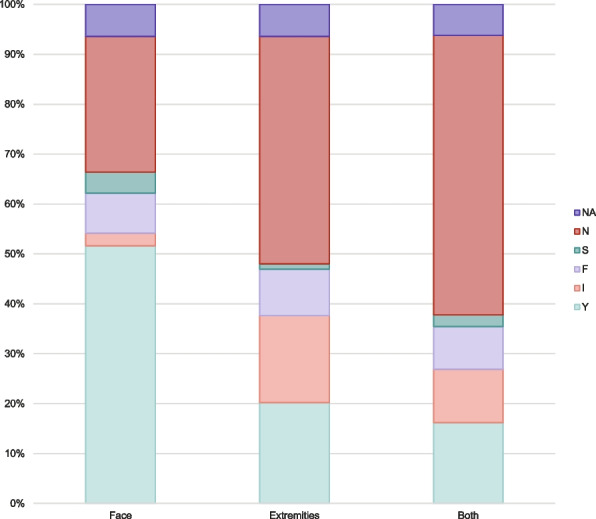
Video examination for facial paresis (face) and motor strength (extremities). “Both” shows the accumulated result, based on the lowest registration between face and extremities. *NA*: not applicable. *N:* not examined. *S*: spontaneous movement/motor deficit on video. *F*: does not follow commands.* I: *incomplete or incorrect examination (attempted). *Y*: yes (exam performed correctly)

The analysis of stroke examination revealed that of the total 550 included calls, 52% of patients were correctly examined for facial paresis (*n* = 284), while 20% were properly examined for extremity paresis (*n* = 111), and 16% received accurate examination for both facial and extremity paresis (*n* = 89). Inadequate examination was noted in 17% of extremity assessments (*n* = 96) compared to just 3% for facial paresis examinations (*n* = 14). Additionally, 9% of patients did not follow instructions for assessment of both face and extremities (*n* = 47) (Fig. 3). Of the correctly or inadequately examined patients (*n* = 387), 90% had a correct positioning (*n* = 357), whereas positioning could not be evaluated in the patients where examination was not attempted (*n* = 153).

The median and mean age was 57. However, for the missed stroke group, the median age was 70, whereas the median age was 37 for the group where stroke was considered but not suspected after examination or diagnosed later.

**Fig. 4:**
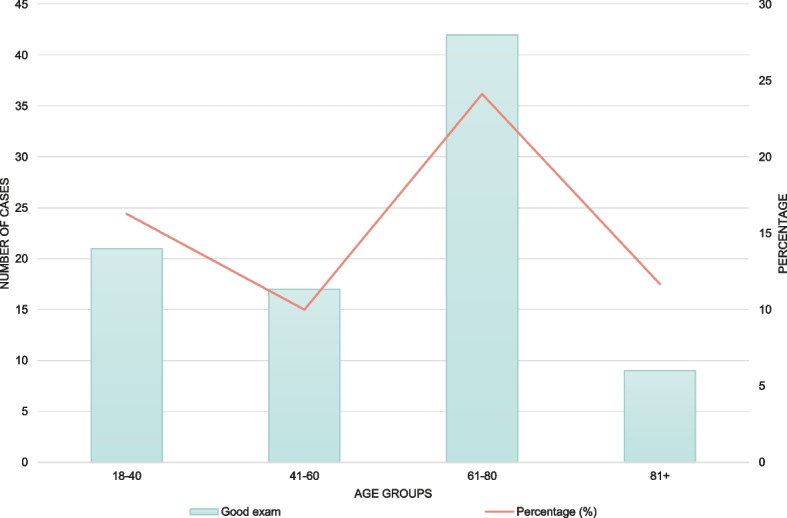
Good examination for facial paresis and motor strength grouped by age category

The bar chart stratified by age category (Fig. 4) indicates that the highest proportion of correct examinations occurred in the 61–80-year age group (24%).

Regarding gender, no statistical difference was found between examination of men and women out of the total dataset. However, regarding missed stroke, men were more sufficiently examined.

As for the patients with a language barrier (*n* = 67), 21% had a stroke reported in the DSR (*n* = 14), of which 71% were a confirmed stroke (*n* = 10) and 29% was a missed stroke (*n* = 4), whereas 79% did not have a stroke according to the DSR (*n* = 53). Out of the total 550 patients, 25% of patients (*n* = 136) were registered in DSR upon arrival at the hospital. The diagnostic distribution was 12.5% haemorrhagic stroke (*n* = 17), 66% ischemic stroke (*n* = 90), 19% TIA (*n* = 26), and 1.5% non-specific stroke (*n* = 2). Out of the 136 patients with stroke, 54% received a stroke criterion from the medical professionals in EMC or OOHS (*n* = 73, confirmed stroke), whereas 46% did not (*n* = 63, missed stroke). However, of the latter group of 63 patients with missed stroke, 49% did receive an urgent ambulance response triaged as A (*n* = 31), and of the remaining 32 patients with missed stroke who did not receive an A response, 5 patients were registered as TIA in the DSR. Consequently, 43% of patients (*n* = 27) of the missed stroke patient group did not receive an A response. In our data, the medical professionals have a sensitivity for detecting stroke with live video at 58% (excl. TIA from the false negative missed stroke) and a positive predictive value at 16.2%.

**Fig. 5:**
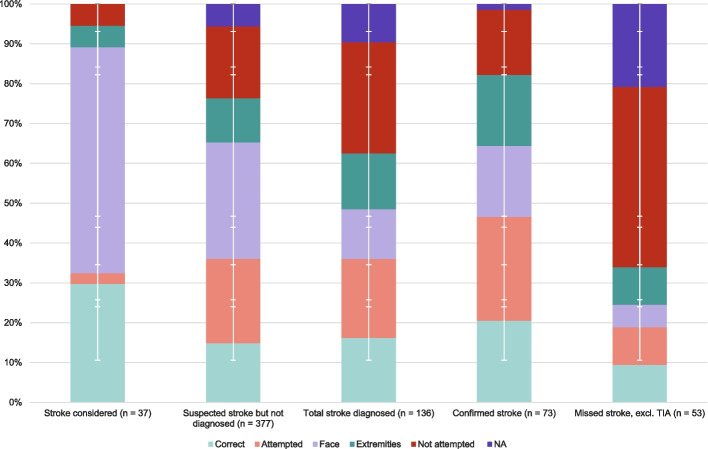
Quality of examination divided by subgroups. *Correct*: Both facial and extremities paresis were adequately examined and positioned correctly. *Attempted:* The patient does not follow commands, incomplete or incorrect examination. *Face:* Facial symptoms were examined without examination for symptoms in the extremities. *Extremities:* Symptoms in the extremities were examined without examination for facial symptoms. *No attempt:* Examination was not attempted. *NA:* The examination analysis was ‘not applicable’ due to affected consciousness

Patients with confirmed stroke undergo significantly more accurate examinations, compared to patients with missed stroke, (*p* < 0.001). Among patients with confirmed stroke (*n* = 73), 21% were correctly examined, 26% had an examination attempted, 18% were examined for facial symptoms without examination of the extremities, 18% were examined for symptoms in the extremities without examination for facial symptoms, and 16% were not examined. Additionally, 1% of patients were deemed not applicable for examination, primarily due to patient unconsciousness. In contrast, for patients with missed stroke excl. TIA patients (*n* = 53), 9% were correctly examined, 9% had an examination attempted, 6% were examined for facial symptoms without examination of the extremities, 9% were examined for symptoms in the extremities without examination for facial symptoms, while 45% were not examined. For this group, 21% were classified as not applicable for examination, once again primarily due to patient unconsciousness (Table 2 and Fig. 5).

**Table 2:** Stroke assessment during video transmission, stratified by whether stroke was suspected and/or later confirmed

*Examination*	Correct	Attempt	Face	Ext.	No attempt	NA
*Suspected stroke but not diagnosed stroke (n* = *377)*	15% (*n* = 56)	21% (*n* = 80)	30% (*n* = 110)	11% (*n* = 42)	18% (*n* = 68)	6% (*n* = 21)
*Suspected stroke and diagnosed stroke (confirmed stroke, n* = *73)*	21% (*n* = 15)	26% (*n* = 19)	18% (*n* = 13)	18% (*n* = 13)	16% (*n* = 12)	1% (*n* = 1)
*Not suspected stroke but diagnosed stroke (missed stroke, n = 53, TIA excluded)*	9% (*n* = 5)	9% (*n* = 5)	6% (*n* = 3)	11% (*n* = 5)	45% (*n* = 24)	20% (*n* = 11)
*Stroke considered, but not suspected after examination and not diagnosed (n* = *37)*	30% (*n* = 11)	3% (*n* = 1)	57% (*n* = 21)	5% (*n* = 2)	5% (*n* = 2)	0% (*n* = 0)

*Correct*: a correct examination. *Attempt*: an attempted examination. *Face:* an examination for facial symptoms without examination of the extremities. *Ext*.: = an examination for symptoms in the extremities without examination for facial symptoms. *No attempt: *an examination was not attempted. *NA*: an examination analysis was not applicable

*Suspected stroke:* the medical dispatcher activated a stroke response

*Diagnosed stroke:* a stroke diagnosis was made at the hospital

*Stroke considered:* the medical professional mentioned stroke or asked about stroke-related symptoms during the call

Furthermore, during the call it was noted whether the medical professional considered stroke, for example if stroke was mentioned or symptoms of stroke were explored. In the confirmed stroke group, the medical dispatcher considered stroke in 84% of cases (*n* = 61) before adding the live video, whereas it was the case for 33% of the missed stroke group (*n* = 21) and 28% in the missed stroke group excl. TIA (*n* = 15). The group where stroke was considered but not suspected after examination and stroke was diagnosed (stroke considered, *n* = 37), had the highest number of correct examinations both in general (30%) and for facial paresis alone (57%).

**Fig. 6:**
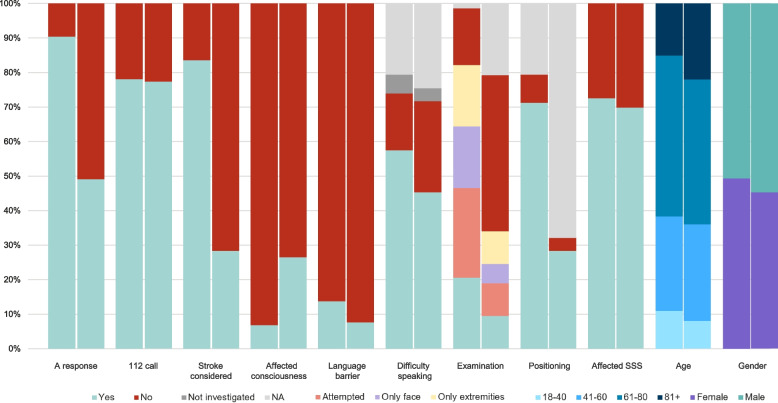
Differences between confirmed stroke and missed stroke (TIA excluded). Comparison of the patients with confirmed stroke (left) and missed stroke (right) for categories regarding the emergency call, potential barriers for the examination, the examination itself, and the clinical presentation at the hospital, as well as age and gender. *NA: not applicable. SSS: Scandinavian Stroke Scale. A response: ambulance with lights and sirens*

A further comparison of the groups reveals notable differences. In the confirmed stroke group, 91% of patients received an urgent response (Category A), compared to 49% in the missed stroke group (excluding TIA, since these may receive a B response according to neurological guidelines). Prior to the addition of live video, stroke was suspected in 82% of confirmed stroke cases, in contrast to 28% of patients in the missed stroke excluding TIA group. Regarding consciousness, 7% of patients in the confirmed stroke group exhibited impaired consciousness, in contrast to 26% in the missed stroke excluding TIA group. Furthermore, 93% of patients with confirmed stroke presented an affected Scandinavian Stroke Scale (SSS) score upon hospital arrival, compared to 70% of the missed stroke excluding TIA group Fig. 6.

**Fig. 7:**
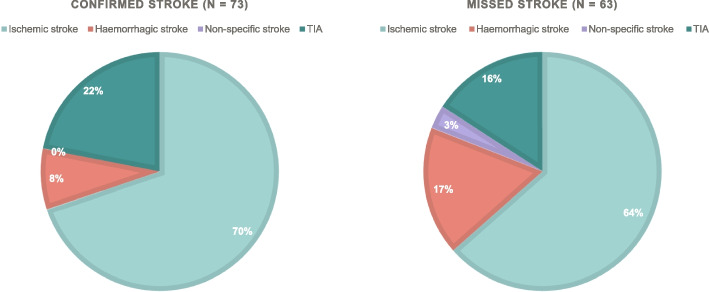
Stroke classification at the hospital. *Confirmed stroke*: The medical dispatcher suspected stroke and stroke was diagnosed at the hospital. *Missed stroke*: The medical dispatcher did not suspect stroke, but stroke was diagnosed at the hospital

The patient group with missed stroke had a diagnosis stratification of 64% with ischemic stroke, 17% with hemorrhagic stroke, 16% with TIA, and 3% with non-specific stroke. In contrast the group with confirmed stroke had a diagnosis stratification of 70% with ischemic stroke, only 8% with hemorrhagic stroke and 22% with TIA (Fig. 7).

**Fig. 8:**
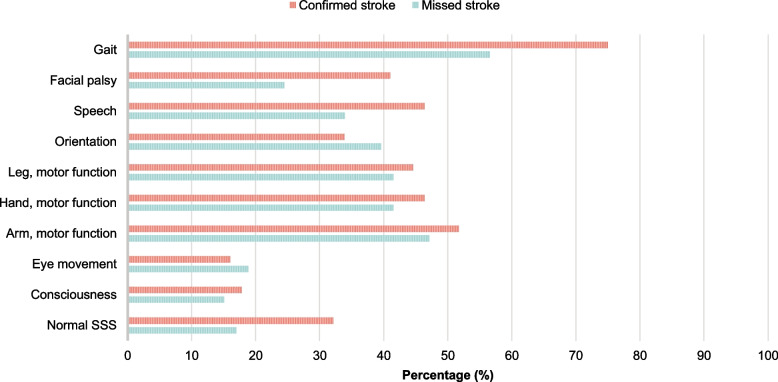
Symptoms of potential stroke upon arrival to the hospital. Potential stroke symptoms were assessed using the Scandinavian Stroke Scale (SSS). *Confirmed stroke*: The medical dispatcher suspected stroke and stroke was diagnosed at the hospital. *Missed stroke*: The medical dispatcher did not suspect stroke, but stroke was diagnosed at the hospital

Furthermore, in this group, upon hospital admission, the positive findings on the SSS were observed in descending order of frequency for the following functional impairments: gait (*n* = 30), arm (*n* = 25) or hand (*n* = 22) or leg (*n* = 22) motor function, orientation (*n* = 21), speech (*n* = 18), facial paresis (*n* = 13), eye movement (*n* = 10), and lastly, consciousness (*n* = 8). As for the patient group with confirmed stroke, similar percentages were found with increased percentages for gait, facial paresis and speech difficulties (Fig. 8).

It is apparent that only 2 of the 10 TIA patients had neurological deficiencies on arrival to the hospital (SSS) and both were due to gait impairment.

## Discussion

Live video is not routinely used for stroke assessment during emergency calls. On average, about 16,000 stroke-related calls occur over a five-year period across the EMCC and OOHS [11]. In our five-year study, video was added in 708 calls, representing roughly 4% of stroke-related calls. However, the addition of live video to stroke-related calls has increased from approximately 1% in 2019 to approximately 13% in 2023. In cases where video is applied, only 16% of patients are sufficiently examined for stroke on video, the majority of which were correctly positioned and within frame. Facial paresis including smiling or showing of teeth was more often assessed that motor paresis of the extremities, and the ‘stroke considered’ group had the highest amount of examined cases. Patients with missed stroke had significantly less accurate examinations and more often hemorrhagic stroke, compared to patients with confirmed stroke.

The sensitivity for stroke detection by the medical dispatchers was 58%, aligning with previous findings in EMCC Copenhagen [11, 23], and among the missed stroke cases excluding TIA only half received an urgent ambulance response. Contrary to our expectations, fewer strokes were detected when live video was used. However, our analyses suggest that live video was preferentially applied in diagnostically more complex cases: stroke was often not suspected prior to video transmission, examinations were less accurate or absent, impaired consciousness was more common, fewer patients had positive SSS findings on hospital arrival, and hemorrhagic stroke was more prevalent compared with confirmed stroke cases. This indicates selection of cases with greater diagnostic uncertainty rather than a reduced diagnostic value of live video. This aligns with previous studies from EMCC Copenhagen, which show that the most frequently assigned criteria codes in unrecognized stroke are diffuse or unspecific codes [24]. Furthermore, the prevalence of a positive SSS score was lower in the missed stroke group, and the most affected parameters were gait disturbance, facial paresis and speech difficulties for both the missed and confirmed stroke group. This is in accordance with previous research, which showed association between aphasia and stroke suspicion in general [25] and that aphasia was the most commonly documented symptom for the group of missed stroke patients [26]. Lastly, the median and mean age in this study was 57 compared to the mean age of 72 of the overall Danish stroke population [27], and therefore elderly patients are underrepresented, possibly due to potential technical difficulties or a medical professional bias, as detected in previous research where older patients were at greater risk of receiving a less urgent emergency response [24].

Technical difficulties were present in few calls, however, the number of calls with technical difficulties may be higher, since an inclusion criterion is that video is added to the emergency call. Video calls have been successful even in suboptimal conditions in previous studies [15], and the proportion of emergency calls with video that could not be established were low (6,1–17,5%) [13, 14, 20, 21], consistent with our findings. Several studies indicate that prehospital live video is a support for clinicians in decision making and increases diagnostic accuracy and triaging decisions [15, 28, 29], Regarding stroke evaluation prehospitally, previous studies have examined telemedicine between a paramedic and a neurologist and found more correct treatment decisions and triaging [29]. However, this is a different setting involving two medical professionals. To our knowledge, the impact of live video from bystanders on medical dispatcher assessment has not yet been investigated.

Video is not frequently used in stroke cases. It may be worth considering whether live video should be implemented when a bystander clearly describes stroke symptoms. However, initiating live video as early as possible could reduce the time spent gathering information, as many details can be directly observed. A study from another region in Denmark found that call duration did not increase with this approach [30]. When considering the use of video, the local context is also important. In Copenhagen, ambulance response times are short; however, given that the positive predictive value is only 16%, implementing a more comprehensive prehospital examination for stroke may reduce unnecessary ambulance dispatches, thereby optimizing resource utilization and reducing costs. As discussed earlier, live video may have been used predominantly in diagnostically more complex cases, emphasizing the need for a structured and comprehensive stroke assessment. One possible approach could be to introduce a structured protocol that includes assessment of facial and limb weakness, gait disturbances, and the presence of aphasia and/or dysarthria. Such a protocol could improve both resource allocation and stroke detection. To ensure quality and facilitate broader implementation, education and training of medical dispatchers will be essential. Nevertheless, live video evaluation by medical dispatchers remains a new field, and best practices have yet to be established.

### Limitations

This group of stroke patients with live video added to the emergency call is not directly comparable to the general stroke recognition at EMC or OOHS, as it is a selected subgroup. The medical dispatchers have not been systematically encouraged in the addition of live video to calls with potential stroke or to note their suspicion of stroke. Therefore, the true negative cases with no stroke response and no stroke diagnosis are unknown, which limits overall diagnostic accuracy and inhibits calculation of specificity and review of these cases. Furthermore, the reasons for adding live video are unknown, and may include challenges such as language barriers, diffuse symptoms or diagnostic uncertainty. Additionally, given that stroke is a condition with serious consequences if missed, a stroke response may have been dispatched even on very low suspicion, potentially leading to overestimation. The medical professionals did not receive specific training in video-guided stroke examination, which may partly explain the higher amount of facial paresis examination, due to its simplicity and time efficacy compared to extremity assessments. Moreover, dispatchers may have stopped the examination once a positive finding was identified, which could contribute to a higher number of incomplete assessments. Visual symptoms are included in criterion A.26.03 (see Appendix 2) but are primarily patient-reported and difficult to assess reliably via live video and were therefore not included in the predefined evaluation criteria for the video analysis. Lastly, the observational retrospective nature restricts ability to infer causality or control for confounding factors, and this study did not examine the perspectives of the medical dispatchers or the bystanders.

## Conclusion

Video is not routinely used for stroke assessment during emergency calls, and a complete and systematic examination for stroke via video was not performed in most patients. Implementation of a structured protocol for stroke examinations during emergency calls with or without live video would be beneficial. However, best practice for use of live video in stroke detection prehospitally is still unknown.

## Data Availability

The datasets generated and analyzed during the current study are not publicly available due to the EU General Data Protection Regulation but are available from the corresponding author on reasonable request from a restricted access server in Copenhagen EMS.

## Appendix 2

### Included EMC and OOHS criteria codes

Danish Index criteria subgroups (in A26: Affected consciousness – paresis – dizziness):

- A.26.03: Potential stroke with presence of sudden facial paresis – and/or decreased strength in arms/legs – and/or speech difficulties – and/or visual disturbances/loss of sight
- A.26.04: Increasingly unconscious – suspected stroke.
- B.26.06: Symptoms of a stroke within the last 24 hours, now asymptomatic.

OOHS criteria codes:

- SS.03.02.01: Transient convulsions/paresis/unconsciousness/unresponsiveness
- SS.03.03: Neurological symptoms with no preceding trauma
- SS.03.03.01: Ongoing or transient aphasia/vision loss/dysarthria
- SS.03.03.02: Ongoing or transient paresis/weakness/numbness of arm and/or leg
- SS.03.03.04: Ongoing or transient paresis/weakness/numbness both arms and/or legs
- SS.03.03.07: Paresis/weakness/numbness of the face and can close the eye
- SS.03.03.09: Double vision (diplopia)
- SS.03.03.11: Slowly progressive paresis/paraesthesia in hand/fingers
- SS.03.07.01: Syncope accompanied by paresis/aphasia/diplopia/chest pain or other neurological or cardiovascular symptoms
